# Early Prediction of the 2019 Novel Coronavirus Outbreak in the Mainland China Based on Simple Mathematical Model

**DOI:** 10.1109/ACCESS.2020.2979599

**Published:** 2020-03-09

**Authors:** Linhao Zhong, Lin Mu, Jing Li, Jiaying Wang, Zhe Yin, Darong Liu

**Affiliations:** 1 Key Laboratory of Regional Climate-Environment for Temperate East AsiaInstitute of Atmospheric Physics, Chinese Academy of Sciences 74521 Beijing100029China; 2 College of Life Sciences and OceanographyShenzhen University 47890 Shenzhen518060China; 3 Shenzhen Research Institute, China University of Geosciences 12564 Shenzhen518057China; 4 College of Marine Science and TechnologyChina University of Geosciences 12564 Wuhan430074China

**Keywords:** Epidemic transmission, infection rate, mathematical model, novel coronavirus, prediction, removal rate

## Abstract

The 2019 novel coronavirus (2019-nCoV) outbreak has been treated as a Public Health Emergency of International Concern by the World Health Organization. This work made an early prediction of the 2019-nCoV outbreak in China based on a simple mathematical model and limited epidemiological data. Combing characteristics of the historical epidemic, we found part of the released data is unreasonable. Through ruling out the unreasonable data, the model predictions exhibit that the number of the cumulative 2019-nCoV cases may reach 76,000 to 230,000, with a peak of the unrecovered infectives (22,000-74,000) occurring in late February to early March. After that, the infected cases will rapidly monotonically decrease until early May to late June, when the 2019-nCoV outbreak will fade out. Strong anti-epidemic measures may reduce the cumulative infected cases by 40%-49%. The improvement of medical care can also lead to about one-half transmission decrease and effectively shorten the duration of the 2019-nCoV.

## Graphical abstract

The flow diagram of early prediction of the 2019-nCoV. The early-stage epidemiological data is firstly analyzed to rule out unreasonable part with considering historical epidemic. The SIR model is then simplified to reduce the number of parameters and thus data requirement. The early prediction is finally obtained via integrating the simplified model with parameter estimations based on objective analysis.

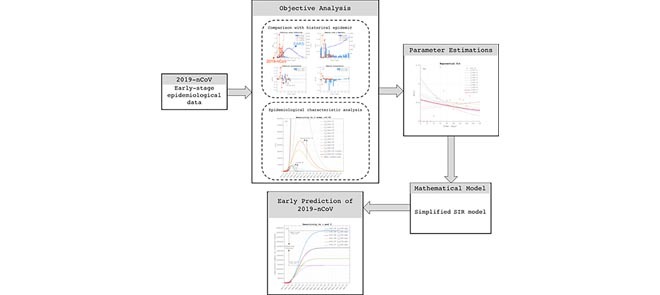

## I. Introduction

**FIGURE 1.**
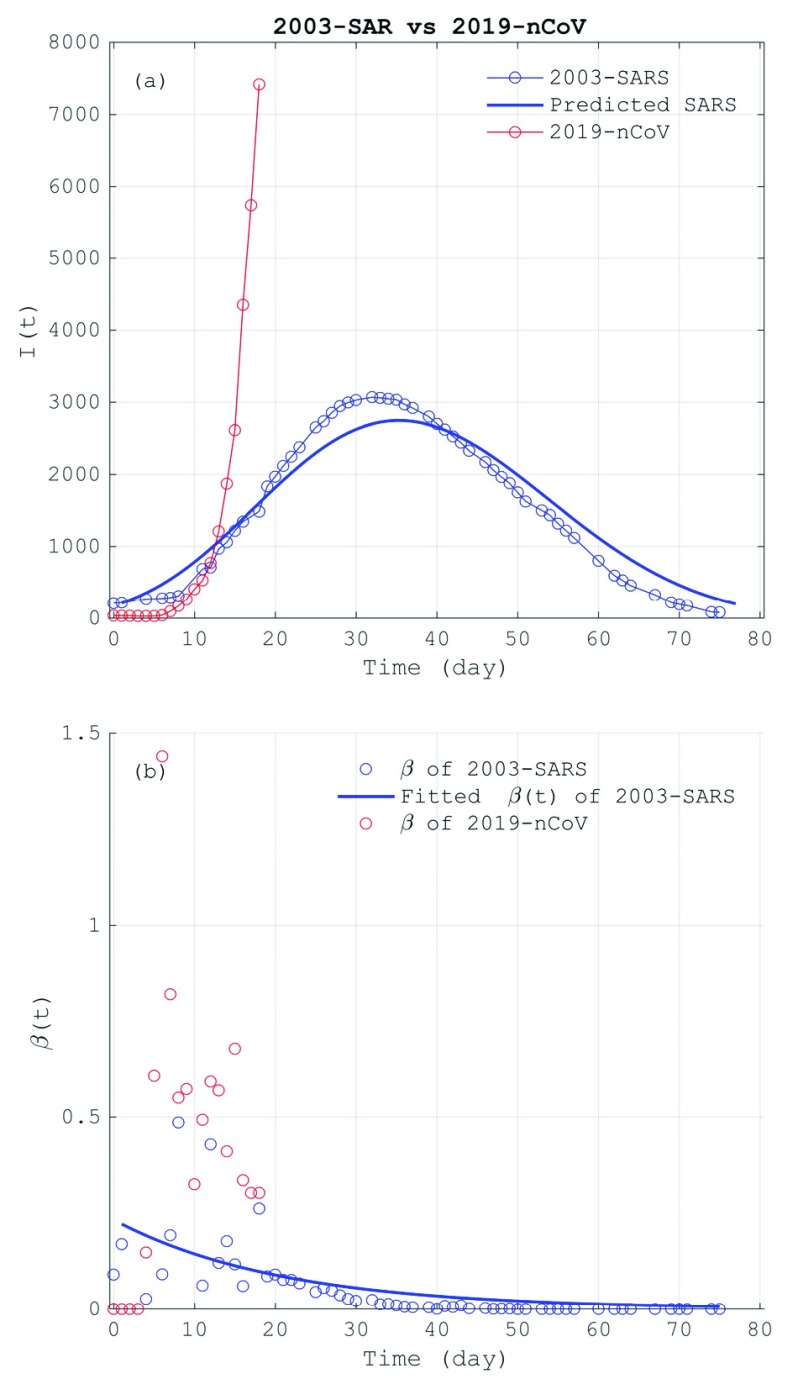
The comparison between SARS in 2003 (2003-SARS) and 2019-nCoV: (a) the number of the infectives (I(t)) of SARS during 10 April 2003 to 24 June 2003 (blue-circle line) and the 2019-nCoV during 11 Jan 2020 to 29 Jan 2020 (red-circle line), where the thick blue line is the prediction of the model Eq. (4) for the 2003-SARS; (b) the infection rates ($\beta $(t)) of SARS (blue circles) and 2019-nCoV (red circles), and the exponentially fitted line of the infection rate of the 2003-SARS (blue line).

Since December 31 2019, the 27 cases of unknown pneumonia were reported in Wuhan City of Hubei Province in South China [1]. On 7 January 2020, Chinese government and the World Health Organization (WHO) identified a novel coronavirus (2019-nCoV) as the causative virus, which belongs to the same virus family of the Severe Acute Respiratory Syndrome (SARS) that outbroke also in South China in 2002–2003 [2]. The 2019-nCoV spread rapidly across most regions in mainland China after 17 January 2020 and leaded to over 7000 infectious cases at the end of January (Fig. 1a). The number of the first-month cumulative cases of the 2019-CoV has exceeded the total number of the SARS cases in 2003, suggesting this novel virus has stronger infectivity than the SARS virus. Since about 23 Jan 2020, the Chinese Government has taken strong measures to prohibit the virus’s transmission, such as warning citizens from going outdoors, temporarily suspending the public transport between some big cities, and even taking quarantine for the main infected city. These unprecedented measures were expected to effectively stop the virus transmission and buy necessary time to deploy medical resources to the affected area. At the same time, considering the virus having exported to other countries, including Thailand [3], Japan [4], South Korea [5] and the United States of America [6], the WHO has made decision on identifying the 2019-nCoV outbreak as a Public Health Emergency of International Concern (PHEIC) [7].

The rapid spread of the 2019-nCoV may be due to multiple causes. One cause is the lacking of information transparency at the early stage of the epidemic outbreak. Releasing the epidemic information in a timely and accurate way is extremely important for the anti-epidemic response of the public. The authentic and transparent information could have prohibited the spread of the 2019-nCoV at the early stage. The other cause is the lacking of scientific diagnostic criterion for the 2019-nCoV. Rapid developing exact testing techniques for a novel virus is very difficult. In fact, the symptoms of the 2019-nCoV are highly similar to those of flu. This aggravated the hardship of diagnosis. The last but not the least, the lacking of an epidemic warning and prediction system lost the opportunity to prohibit the epidemic spread at the initial stage.

Under the background of the ongoing 2019-nCoV transmission, the epidemiological survey is extremely important for stopping transmission by tracing the infectious pathways and particularly identifying the super spreaders. But, the outbreak of the 2019-CoV is just coincident with the large-scale population migration during the most important traditional Chinese festival, which exacerbated the spreading of virus and simultaneously greatly increased the difficulty of the epidemiological investigation. So, on the other hand, monitoring and predicting the evolution of the epidemic becomes extremely meaningful for the decision making against the public health crisis. Mathematical modelling has gained more attention and awareness in epidemiology and the medical sciences [8]–[10]. One family of these models is the dynamical epidemic model called Susceptible-Infected-Removed (SIR) model [11]. The SIR model originated from the study of the plague almost one hundred years ago [12]. Tremendous advance has been achieved in dynamical epidemic model since mid-20^th^ century [13]. In recent decades, some realistic factors influencing the epidemic transmission were included in the classic SIR model, such as the model considering the incubation stage [14], the SEIRS model considering the population age and the population exposed to epidemic [15], and the SIS model including birth and death of the susceptible [16]. Some dynamical models were also designed for specific epidemic. For example, the dynamical models were designed to simulate spreading of HIV, SARS and Middle East Respiratory Syndrome (MERS) [2], [17]–[19]. As the development of new methods, the complex network and machining learning were combined with the dynamical model and created a promising direction of the epidemic prediction [20], [21].

The construction of the SIR model for an epidemic disease needs determining several key parameters (e.g. the infection rate and the removal rate) empirically or statistically. As was mentioned in the above, the family of the SIR model has developed complex model to considering more detailed classes of individuals, such as the exposed class and the protection class [11]. In general, the more realistic the model is, the more precisive prediction the model can make. But, the model considering more realistic factors needs more data to define additional parameters. On the other hand, some effort has also been made on simplified model. For example, the well-known logistic equation was used of as the two-compartment “S-I” (Susceptible -Infectious) model, which can be further included some stochastic processes in the modelling of SARS [22]. The above-mentioned researches prove that the SIR-family models at different complex levels can well capture the basic mechanism of the epidemic transmission.

In this study, we tried to present an early prediction of the epidemic of the 2019-nCoV based a simplified SIR model. The rationality of the available epidemiological data was analyzed firstly so as to obtain the reasonable estimation of the key parameter, i.e., infection rate. Focusing on the infection rate and removal rate, several experiments were designed to simulate the spreading of 2019-nCoV under different levels of anti-epidemic measure and medical care. The prediction intervals of the infective number and its inflection point, as well as those of the cumulative infected cases and the fading-out time of the epidemic, were presented. Our results are supposed to provide important information for the crisis management against the novel coronavirus.

## II. Data and Methods

### A. Data

The 2019-nCoV data used in this study has several sources, including: (1) Wuhan Municipal Health Commission (http://wjw.wuhan.gov.cn/), providing infective data in Wuhan from December 31, 2019 to January 19, 2020; (2) Health Commission of Hubei Province (http://wjw.hubei.gov.cn/), providing the latest epidemiological data in Hubei Province from January 20, 2020 up to the present, including cumulative confirmed cases, deaths, suspected cases, and cures in cities of Hubei Province; (3) National Health Commission of the People’s Republic of China (http://www.nhc.gov.cn/), providing the latest epidemiological data of China from January 20, 2020 up to the present. The data of SARS is obtained from WHO (https://www.who.int/). It provides epidemiological data all over the world from March 17, 2003 to July 11, 2003 [23].

### B. Simplified SIR Model

Under the assumption of no change of population due to other causes and considering a disease that confers immunity after recovery, we can divide the population into three distinct classes: the susceptibles (S), namely the healthy population vulnerable to infection; the infectives (I), the infected population; and the removed infectives (R), the population has no transmissibility including the recovered and dead infectives. The population of the three classes is governed by the following system of nonlinear ordinary differential equations [19]:

\begin{align*} dS/dt=&-\beta S\left ({t }\right)I\left ({t }\right) \tag{1}\\ dI/dt=&\beta S\left ({t }\right)I\left ({t }\right)-\gamma I\left ({t }\right) \tag{2}\\ dR/dt=&\gamma I\left ({t }\right)\tag{3}\end{align*}

where $t$ is time (in day); $\beta $ the infection rate, i.e., the infected ratio by one infective during unit time; and $\gamma $ the removal rate, i.e., the ratio of the removed number to the number of the infectives. Equations (1-3) are coupled through the two right-hand-side (RHS) terms: $\beta S\left ({t }\right)I\left ({t }\right)$, i.e., the newly increased infectives; and $\gamma I\left ({t }\right)$, i.e., the newly removed infectives. For Eq. (1), the solution of $S$ generally follows a function monotonically decreasing to a stable final value until the epidemic fading out [11]. The initial value of $S$ is given empirically according to the community population affected by the disease. But, the 2019-nCoV outbroke in Wuhan, a big city with population over ten million. The high population density and frequent population mobility made it hard to exactly estimate the susceptible population in Wuhan, let alone the whole mainland China. Therefore, we assume that the infected population of the 2019-nCoV can be omitted with compared to the huge susceptible population in China. That is to say, we can treat the variable $S$ as a large constant with compared to the variable $I$. With this assumption, Eq. (1) can thus be dropped out from the coupled system Eq. (1-3). For the rest two-equation system, i.e., Eqs. (2-3), the change of the infectives ($I$) is thought to be more important for epidemic prediction than the removed infectives ($R$). So, we can further drop Eq. (3) and lead to a single-equation system, i.e., Eq. (2). In the finite-difference form, Eq. (2) can be discretized as

\begin{equation*} I\left ({t+\mathrm {\Delta }t }\right)=I\left ({t }\right)+\left ({\beta -\gamma }\right)I\left ({t }\right)\mathrm {\Delta }t,\tag{4}\end{equation*}

where $\Delta t$ is the time interval of numerical integration, and the constant $S$ is combined into the infection rate $\beta $ with the definition of

\begin{equation*} \beta \left ({t }\right)=\left [{ I\left ({t+\mathrm {\Delta }t }\right)-I\left ({t }\right) }\right]/\left [{ I\left ({t }\right)\Delta t }\right]\tag{5}\end{equation*}

And, the removal rate $\gamma $ is similarly defined as

\begin{equation*} \gamma \left ({t }\right)=\left [{ R\left ({t+\mathrm {\Delta }t }\right)-R\left ({t }\right) }\right]/\left [{ I\left ({t }\right)\Delta t }\right]\tag{6}\end{equation*}

In Eq. (4), the two parameters, $\beta $ and $\gamma $, need to be set before performing model prediction. In real epidemic transmission, the infection rate $\beta $ is a time-varying variable that can be statistically estimated via fitting the epidemiological data. In principle, the parameter $\gamma $ can be also estimated in the similar way. Equation (4) with the definitions of $\beta $ and $\gamma $ can be temporally integrated forward. Here, we use MATLAB to realize the simple numerical computation of Eq. (4). Based on the prediction of $I$, the removed infectives can be obtained by $\gamma I\left ({t }\right)$. With the numbers of $I $ and the removed infectives, we can further obtain the cumulative number of infected cases by simply summing $I\left ({t }\right)$ and $\gamma I\left ({t }\right)$.

### C. Method of Parameter Estimation

As was mentioned in previous section, the 2019-nCoV and SARS belongs to the same family of coronavirus. Here, we assume the two kinds of viruses both follow the basic rule of epidemic transmission. From the knowledge of epidemic transmission [22], the variable $I$ follows a bell-shaped function, i.e., increasing from zero to a turning point and then decreasing to a stable value; the variable $S$ and $R$, respectively, follow the monotonically decreasing and increasing functions until reaching their stable states. This requires the parameter $\beta $ and $\gamma $ satisfying a monotonically decreasing and increasing function, respectively. So, this is one key criterion to fit the parameters in practice.

The infection rate ($\beta$) and removal rate ($\gamma$) can be obtained statistically or empirically. For the early prediction discussed here, we should firstly evaluate the reliability of the available data with combing the knowledge of the historical epidemic, such as SARS in 2003. To do this, several subsets of the epidemiological data are extracted by resampling from the available data. We tried to identify the reasonable subsets of epidemiological data for the model prediction via evaluating the reasonability of the fitted infection rate ($\beta$) from different subsets of data. In the objective analysis of data, there are two criterions used to rule out the unrealistic dataset. One is that the fitted parameter $\beta $ should be monotonically decreasing with time, otherwise, the modelled epidemic will not stop unless all the susceptibles become infectives. This extreme situation is particularly impossible for the epidemic spread over an area like China, which has large spatial scale and huge susceptible population. The other rule-out criterion is the fitted $\beta $ exhibits unrealistically sharp decrease, which will let model significantly underestimate the epidemic transmissibility and predict much less infectives than the real data. Through investigating the characteristics of the fitted infection rates based on different data subsets, we can obtain the reasonable estimates of $\beta $ according to the above two criterions.

For the parameter $\gamma $, it generally varies slowly at the initial stage of the epidemic outbreak because most infectives have not yet reached the recovery stage. So, the fitted $\gamma $ based on the early epidemiology data is bound to be underestimated, and thus cause unrealistically long duration of the epidemic spread. Thus, we here treated the parameter $\gamma $ as a constant with referring to the removal rate computed from the real data.

After we obtaining the reasonable ranges of the two parameters, we can out sensitivity experiments with respect to the parameter intervals to find the prediction intervals of the infectives and associated variables.

## III. Results

Although the 2019-nCoV is a member of the coronavirus, the 2019-nCoV still shows some different characteristics from SARS. Figure 1a shows the number of infectives ($I$) and the SIR prediction of the SARS over Mainland China during 10 April 2003 to 24 June 2003 [23]. The simplified SIR model (Eq. (4)) well depicts the propagation of the SARS in 2003. In contrast, the 2019-nCoV shows a pulse-like increase of infected cases after day 10. As shown by Table 1, the epidemiological data that can be used by the model was released since 11 January 2020 although some cases of unknown pneumonia have been identified earlier. During 11 January −15 January, the cumulative infectives keeps unchanged at 41 cases, i.e., zero infection rate ($\beta$) in the first 5 days. But, on 18 January, the infectives (121 cases) is almost doubled, which causes a pulse-like infection rate of 1.44 per day. The huge change of the infection rate seems not to be explained as the natural variation of the epidemic. The sharp increase of the confirmed infected cases at such a short time may be attributable to the improvements of the government emphasis on the disease and the diagnosis technique. Therefore, the data before 18 January should be used with caution. Since then, all the provinces of China began to continuously release and update the epidemiological data. It is supposed that the data after that day is more reliable to reflect the characteristic of the 2019-nCoV.

**TABLE 1:** The Epidemiological Data of the 2019-nCoV: the Cumulative Number of the Infected Individuals (I), the Cumulative Removed Infectives (R), the Infection Rate ($\beta$) and the Removal Rate ($\gamma$). The Removed Infectives Include the Dead and Cured Infectives

Date	Cumulative I(t)	Cumulative R(t) (Dead+Recovery)	$\beta (t)$	$\gamma (t)$
2020-01-11	41	7(1+6)	0	0
2020-01-12	41	8(1+7)	0	0.03
2020-01-13	41	8(1+7)	0	0
2020-01-14	41	14(2+12)	0	0.18
2020-01-15	41	14(2+12)	0	0
2020-01-16	45	17(2+15)	0.15	0.11
2020-01-17	62	21(2+19)	0.61	0.14
2020-01-18	121	27(3+24)	1.44	0.15
2020-01-19^1^	198	29(4+25)	0.82	0.02
2020-01-20^2^	291	31(6+25)	0.55	0.01
2020-01-21	440	37(9+28)	0.57	0.02
2020-01-22	571	45(17+28)	0.32	0.02
2020-01-23	830	59(25+34)	0.49	0.03
2020-01-24	1287	79(41+38)	0.59	0.03
2020-01-25	1975	105(56+49)	0.57	0.02
2020-01-26	2744	131(80+51)	0.41	0.01
2020-01-27	4515	166(106+60)	0.68	0.01
2020-01-28	5974	235(132+103)	0.34	0.02
2020-01-29	7711	294(170+124)	0.30	0.01

1. Statistics before January 19, 2020 are sourced from the Wuhan Municipal Health Committee.

2. Statistics after January 20, 2020 are sourced from the National Health Committee of the People’s Republic of China.

As shown by Table 1 and Fig. 1b, the infection rate of the 2019-nCoV is over 0.5 per day in most of time after 17 January. That means every two 2019-nCoV infectives can infect at least one person per day. This rate is more than the doubled infection rate of the SARS in 2003 (Fig. 1b). Therefore, from the initial epidemiological characteristics, the 2019-nCoV virus exhibited a much stronger infectivity than the SARS virus. This feature was also reported by some latest model studies of 2019-nCoV [24], [25]. As was mentioned in the above, the infection rate is a key parameter determining the prediction of the epidemic spread based on the SIR model. In order to predict the future infective number, the infection rate is often specified as a constant or a time-varying analytical function. For SARS, 2019-nCoV and other epidemics, treating the infection rate as a time-varying variable [26] can capture the dynamical process of the epidemic transmission, including the natural processes and the human intervention. As the fitted line shown in Fig. 1b, the infection rate of the SARS in 2003 well satisfies a monotonically decreasing exponential function, particular after day 20. This analytical function of $\beta $ is then substituted into Eq. (4) to make epidemic prediction. Figure 1a shows that the predicted the evolution of the infective number of 2003-SARS is highly consistent with the real data (Fig. 1a).

**FIGURE 2.**
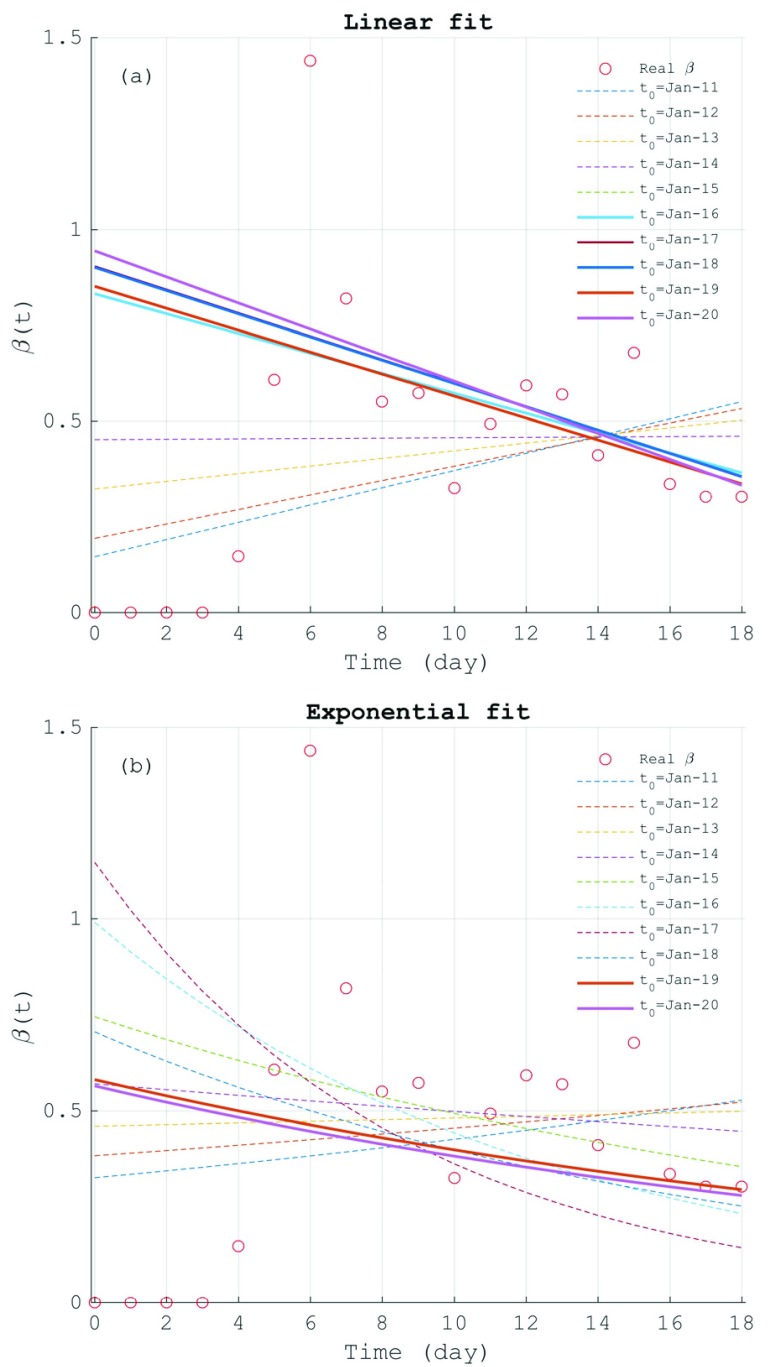
The infection rate of the 2019-nCoV: (a) the infection rate ($\beta $(t)) of the 2019-nCoV (red circles) and the linearly fitted lines; (b) the same as (a) but for the exponentially fitted infection rate with the unrealistic fitting curves dashed.

For the 2019-nCoV, considering the poor quality of the epidemiological data of the 2019-nCoV before 18 January, we extracted several subsets from the available epidemiological data shown in Table 1 via sequentially excluding the data between 11 January to 20 January in one-day interval. Thus, we can obtain the ten subsets successively named by the first day of the extracted data, i.e., $t_{0}=\text {Jan}-11$, $t_{0}=\text {Jan}-12,\ldots, t_{0}=\text {Jan}-20$. The dataset $t_{0}=\text {Jan}-11$ means all of the released data since 11 January is extracted, and $t_{0}=\text {Jan}-20$ for the data after 19 January. These subsets of data are then used to fit $\beta $ functions of the 2019-nCoV. Therefore, we totally got 10 linear functions and 10 exponential functions of $\beta $, as shown in Fig. 2. Ten straight lines in Fig. 2a are those fitted by linear regression model. It is found that the positive slopes of the fit lines are obtained based on the datasets before 16 January. After that day, the negative slopes for $\beta $ are fitted. With the knowledge of the historical epidemic, the parameter $\beta $ has to be a constant or satisfy a monotonically decreasing function. The increasing $\beta $ will lead to unrealistic growth of the infectives that cannot converge to a stable state. So, the data before 16 January should be ruled out from the analysis. The rest five linear functions, namely from $t_{0}=\text {Jan}-16$ to $t_{0}=\text {Jan}-20$, can be substituted into the model Eq. (4) for temporal integration.

**FIGURE 3.**
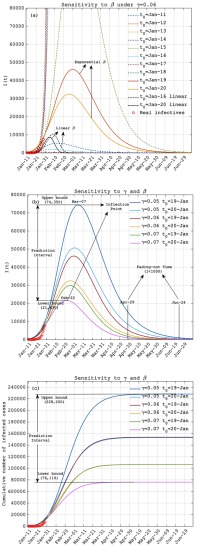
The prediction of the infected cases of the 2019-nCoV under different infection rate functions (($\beta \text{t}$)) and removal rate ($\gamma $): (a) the sensitivity of the number of the infectives ($I(t))$ to the fitted functions of ($\beta \text{t}$) in Figs. 2 under moderate removal rate ($\gamma =0.06$), where the unreasonable results are dashed. (b) the same as (a) but for sensitivities to exponential $\beta $ functions ($t_{0}=\text {Jan}-19,\,\,\text {Jan}-20$) and $\gamma $ ($\gamma =0.05, 0.06, 0.07$). (c) the same as (b) but for the cumulative number of infected cases. The red circles in each plot is the real data released by the Chinese Government since 11 Jan 2020.

On the other hand, the above results of 2003-SARS (Fig. 1b) show the parameter $\beta $ well satisfies a slowly decreasing exponential function. Similarly, we also tried to exponentially fit the 2019-nCoV infection rate. As shown by Fig. 2b, the fitted $\beta $ exponential functions also show large differences between different subsets of data. The uncertainties are largely caused by the data quality. It was found that all the fitted functions using the data before 14 January ($t_{0}=\text {Jan}-11$ to $t_{0}=\text {Jan}-13$) are monotonically increasing, which can be ruled out according the above-mentioned criterions. The estimations of $t_{0}=\text {Jan}-16$ to $t_{0}=\text {Jan}-18$ have steep downward slopes due to the pulse-like increase of $\beta $ between 17 Jan-18 Jan, which may result in unrealistically strong prohibition of the infective number. On the contrary, the unrealistic increase of the predicted infectives may be caused by the $\beta $ function with slow decreasing speed ($t_{0}=\text {Jan}-14$) or large base infection rate ($t_{0}=\text {Jan}-15$). Only the two $\beta $ functions ($t_{0}=\text {Jan}-19$ and $t_{0}=\text {Jan}-20$) seems to have appropriate slopes and base values. The reasonability of the $\beta $ functions from subset $t_{0}=\text {Jan}-14$ to $t_{0}=\text {Jan}-20$ will be further discussed in Fig. 3a.

Recalling the simplified SIR model (Eq. (4)) is jointly determined by $\beta $ and $\gamma $. So, we also computed the values of the removal rate ($\gamma$) based on the available data. As shown in Table 1, the mean value of $\gamma $ is about 0.04, which means four out of one hundred infectives are cured or dead per day. It is also found that the parameter $\gamma $ stays around 0.02 with weak decreasing trend after 17 January, but fluctuates in large amplitude between 0.1 and 0.2 before that day. This dramatic change of the removal rate also reflects the unnatural influence mentioned above. But for the data after 17 January, the low removal rate with the decreasing trend may be attributable to that most infectives have not yet finished the whole course of the disease. So, at the initial stage of the epidemic outbreak, few fully recovered or dead infectives is not enough to exactly fit the function of the removal rate. The slightly downward trend of $\gamma $ (Table 1) will lead to a monotonically decreasing function and thus extreme overestimation of the infected cases by model prediction. So, instead of setting a varying removal rate, this work specifies $\gamma $ as a constant. The sensitivity experiments (figure not shown) to $\gamma $ were performed under the two $\beta $ functions ($t_{0}=\text {Jan}-19$ and $t_{0}=\text {Jan}-20$) (Fig. 2b). With compared to the available real data, the hindcasting infective number is obviously overestimated under $\gamma < 0.03$ but underestimated under $\gamma >0.09$. So, we set $\gamma $ as 0.05-0.07 to keep it away from the two threshold values.

Using the $\beta $ functions fitted in Fig. 2 and the constant $\gamma $, we can further perform the model prediction. In order to simplify the problem, we only select the two linear $\beta $ functions, i.e., the line with minimal ($t_{0}=\text {Jan}-16$) and maximal ($t_{0}=\text {Jan}-20$) negative slopes in Fig. 2a, to represent the change interval of the linear functions of $\beta $. In addition, all the $\beta $ functions shown in Fig. 2b are substituted into Eq. (4). Figure 3a demonstrates the predictions of the infective number ($I$) based on the $\beta $ functions shown in Fig. 2 with moderate removal rate ($\gamma =0.06$). For the exponentially increasing ($t_{0}=\text {Jan}-11$ to Jan-13) and too weakly decreasing ($t_{0}=\text {Jan}-14)\,\,\beta $ functions, too strong infection rate will cause the exponentially increase of the infected cases, i.e., unstable solution, an extremely unrealistic prediction. For But for the $\beta $ functions with too steep downward slopes ($\text{t}_{0}=\text {Jan}-16$ to Jan-18), the model predicts unreasonably low infected cases, which is much lower than that from the contemporaneous real data. The above discrepancies are mainly caused by some unrealistically high infection rates due to pulse-like increase during 17–18 January (Table 1). For the same reason, the $\beta $ function of Jan-15 is also ruled out from the model prediction to completely eliminate the influence of the false data on the infection rate although the predicted result seems reasonable (Fig. 3a).

For the linear $\beta $ functions in Fig. 2a, the corresponding prediction interval shows unrealistic low infected cases, whose peak value is only slightly higher than the real infective number at the initial stage of 2019-nCoV. The predicted duration (30-40days) of the epidemic spread also seems too short with compared to the 2003-SARS, which shows lower infection rate but longer duration (about 70 days) (Fig. 1a). Thus, we can infer that the linear regression model is an inappropriate model for fitting the infection rate of 2019-nCoV.

With the above discussion, we finally retained only two experiments, i.e., those including $t_{0}=19-\text {Jan}$ and $t_{0}=20-\text {Jan}$ exponential $\beta $ functions from Fig. 2b. In fact, these two experiments, respectively, correspond to the high- ($t_{0}=19-\text {Jan}$) and low- ($t_{0}=20-\text {Jan}$) infection-rate experiments, which can also be understood as the low- and high-level anti-epidemic measures against the virus transmission in model simulation, respectively. Under the moderate value of $\gamma $ (Fig. 3a), the infected of 2019-nCoV will reach its peak value in late February to early March and fade out completely in late May. The anti-epidemic measure seems to have on significant influence on the fading-out date but have strong effect on the number of the infected cases. The peak of the infected cases may reach 3,2000 under high-level prevention measure, but 46000 under the low-level measure. From Fig. 3c, the strong anti-epidemic measure can reduce about 44% cumulative infected cases under the moderate removal rate.

With the truth that the death cases account for only small part of the removed infectives, the change of the removal rate can be approximately attributed to the factors against virus replication inside human body, such as the improvement of the medicine or therapy. Here, we set the levels of removal rate as $\gamma =0.05, 0.06, 0.07$ to represent low, moderate and high medical levels. Combined with the two anti-epidemic levels defined by $\beta $ function shown in Fig. 2a, i.e., $t_{0}=19-\text {Jan}$ and $t_{0}=20-\text {Jan}$ of exponential $\beta $ functions, we totally get six scenarios of the 2019-nCoV propagation in Figs. 3b, c. With compared to low-level anti-epidemic scenarios ($t_{0}=19-\text {Jan}$), the strengthened measures ($t_{0}=20-\text {Jan}$) reduce the peak infected cases by 47%, 42% and 38%, and the cumulative cases by 49%, 44% and 40% under low, moderate and high medical levels ($\gamma$), respectively.

It is also found that the decrease of the infection rate cannot significantly influence the duration of the 2019-nCoV outbreak under the same $\gamma $. However, the increase of $\gamma $ is more effective in prohibiting the infected number. It is estimated from the model predictions that every increase of the removal rate in $\Delta \gamma =0.01$ (about 16%-20%) will lead to 50%-60% decrease of infected cases (Fig. 3b), and 40%-50% decrease of cumulative cases (Fig. 3c). Furthermore, large removal rate or high-level medical care significantly shortens the duration of the epidemic outbreak. Under the $t_{0}=19-\text {Jan}$ function of $\beta $, the model with $\gamma =0.06$ predicts the 2019-nCoV will fade out in late May. But under $\gamma =0.07$ and the same $\beta $ function, that fading-out time is advanced to early May (Fig. 3b). This reflects the tendency of the epidemic is highly sensitive to the medical-service level. High-level medical care can significantly prohibit the propagation of the epidemic situation.

Considering all the sensitivity experiments with respect to $\beta $ and $\gamma $, the prediction intervals of the 2019-nCoV are concluded in Table 2. The inflection point of the infected case variation is a key indicator for the epidemic transmission monitor. In theory, the inflection point of $I(t)$ can be obtained by simply setting $\text{d}I/\text{d}t=0$ in Eq. (2), i.e., the time point satisfying $\beta S(t)=\gamma $. Recalling the infection rate is a monotonically decreasing function, the turning point of $I(t)$ is thus determined by the variation of the magnitude contrast between the infection rate and the removal rate. From the experiments under different sets of $\beta $ and $\gamma $ (Fig. 3b and Table 2), the inflection point of 2019-nCoV will occur in late February to early March, when the number of the unrecovered infectives will reach its peak value of about 43,000 cases with the variation interval between 22,000 and 74,000. After the inflection point, the number of the infected cases will decrease rapidly until the epidemic has faded out in late April to late June. On the whole, the 2019-nCoV epidemic may persist three to five months. From Fig. 3c and Table 2, the final cumulative infected case will reach about 140,000 varying in the interval of 76,000-230,000, which reflects three-fold difference between the most optimized measure and the worst one. That is to say, the above prediction intervals are strongly determined by the anti-epidemic measures and the medical-service level against the 2019nCoV.

**TABLE 2:** The Prediction Intervals of the 2019-nCoV. The Mean Model Prediction With its Lower and Upper Bounds are Shown. The Mean Prediction is the Arithmetic Mean of the Experiments Shown in Figs. 3b, c. The Date and the Infective Number at Inflection Point are Listed in the 2^nd^ -3^rd^ column. The 4^th^ and 5^th^ Column, Respectively, Show the Date and the Cumulative Number at the Fading-Out Point, Which is Defined as the Infected Population Lower Than 1,000

Prediction	Inflection Point		Fading-out Point	
	Date	Peak Infectives	Date	Cumulative infectives
Lower bound	22-Feb	21,630	29-Apr	76,110
Upper bound	07-Mar	74,350	24-Jun	228,200
Mean	01-Mar	47,990	27-May	152,155

## IV. Discussion

It is hard to accurately predicting the epidemic evolution based on limited data, especially in the condition of lacking reliable data. Although the 2019-nCoV outbreak can be traced back to late December 2019 (perhaps earlier), the systematically released epidemiological data is only available after 11 January 2020, among which the reasonability of the data before 18 January 2020 is still unconvinced. So, until the authors finished this work, the reliable data only covers no more than two weeks, which may lead to large uncertainty in the early prediction of the 2019-nCoV outbreak. But, the effort of the early prediction is still meaningful. Mathematical models of different complexities have been proved to be effective in predicting the evolution of epidemic outbreak. But, the more complex the structure of the model, the more parameters are needed to be determined. Under the condition of lacking epidemiological data, it is hard to objectively determine all the parameters. Too many unknown parameters will bring large uncertainties in the model prediction. Based on the point, this work formulated a simplified SIR model with the least parameters, i.e., the infection rate and the removal rate, to reduce the uncertainty as much as possible. The model shows good ability in hindcasting the spreading of SARS in 2003. So, we further applied this model to the 2019-nCoV.

Through eliminating the unreliable data via objective analysis, we provided epidemic predictions under different scenarios with respect to different-level anti-epidemic measure and medical care represented by the two model parameters, i.e., infection rate and removal rate. The predictions are supposed to be a helpful guide to the decision making in coping with the ongoing 2019-nCoV transmission in China. The strictness of the current quarantine measures and infection control precautions employed by Chinese Government is historically unprecedented. So, as predicted by this work, the control measures should pay more attention to the medical-service aspects, such as accelerating the diagnostic speed and enhancing the hospitalization capacity. If all the above efforts get the cumulative infected cases down to below about 80,000 until late February (Fig. 3c), the severity of the 2019-nCoV may be controlled at the relatively low level finally. The sensitivity of the simplified-model prediction to the parameters also emphasized the importance of the openness and transparency in releasing the data relevant to the public health. As the progressing of the 2019-nCoV, more epidemiological data will be available to verify and revise this early prediction of the 2019-nCoV.
